# Editorial Courtesy

**Published:** 1852-06

**Authors:** 


### THE MEDICAL EXAMINER.

PHILADELPHIA, JUNE, 1852.

### EDITORIAL COURTESY.

The editors of the Stethoscope, published at Richmond, Virginia, and the New Jersey Medical and Surgical Reporter, will please accept our thanks for their kindness in sending us proof sheets of the proceedings of the American Medical Association. Such courtesies are the more grateful from their rarity in these days of selfishness. We trust it may be in our power to reciprocate them.

